# Editorial: Hypotheses explaining the allergy epidemic

**DOI:** 10.3389/falgy.2026.1797554

**Published:** 2026-02-24

**Authors:** Nikolaos G. Papadopoulos, Glenis K. Scadding, Linda Cox

**Affiliations:** 1 Allergy Department, 2nd Pediatric Clinic, National and Kapodistrian University of Athens, Athens, Greece; 2 Lydia Becker Institute, The University of Manchester, Manchester, United Kingdom; 3 Department of Allergy and Rhinology, Royal National ENT Hospital, London, United Kingdom; 4 Nova Southeastern University, Fort Lauderdale, FL, United States

**Keywords:** allergy epidemic, allergy epidemiology, environment, health impact, hygiene

The epidemiology is clear: allergic conditions, as we understand them today, appeared as rare “upper-class” diseases approximately two centuries ago (1). As early as the late 19th century, there were discussions and attempts to explain their apparent epidemic increase (2). This Research Topic brings together the most influential hypotheses proposed to explain the allergy epidemic and investigated over the last 35 years, along with several novel, potentially groundbreaking ideas.

The majority of these hypotheses focus on environmental exposures—or lack thereof. While microbial exposures currently prevail as the leading explanation, changes in lifestyle, climate, dietary habits, pollution, and industrial products may also have contributed to the increase in allergies. The common denominator of these hypotheses is the interaction between the exposome and the immune system. Thus, this Research Topic has implications beyond allergies, offering insights into the broader effects of human-driven environmental change on present and future health.

The starting point is the classic “hygiene hypothesis,” based on Strachan's seminal 1989 article, “*Hay fever, hygiene and household size”* (3). Perkin and Strachan described firsthand the conception of that idea and its evolution over the following 30 years. The original epidemiological observation that British children from larger families were less likely to develop hay fever led to the suggestion that early exposure to infection may prevent allergies. Studies of children from large families with early farming exposure also showed a major reduction in the prevalence of allergic diseases, as did studies of anthroposophic families (4). The authors conclude that the mechanism remains obscure. Nevertheless, over the years, several aspects have been explored, ranging from respiratory infections to microbiomes. For example, a recent study concluded that “the protective effect of older siblings on the risk of developing IgE-mediated food allergy during infancy is substantially mediated by advanced maturation of the gut microbiota at age 1 year” [SIC] (5). Thus, the external microbial environment interacts with and influences the internal microbial environment, both of which are closely connected to immune health.

The link between the external and internal microbial environment—the microbiome—and immune health is the central idea behind the “immunological old friends” concept (6), explored here by its original proponent, Rook. The “old friends hypothesis” postulates that microorganisms drive immune system development and the expansion of immunoregulatory components such as regulatory T cells. Evolutionary studies suggest that the relevant organisms (“Old Friends”) emanate from our immediate environment and are symbiotic components of a healthy microbiome, co-evolving with mammals for millions of years. These microorganisms may disappear due to changes in the external environment, antibiotic use, sterilization, and related practices. This understanding supports the development of measures such as urban planning, diet, and education to correct the immunoregulatory deficit.

A similar concept, although focused more on the ecological characteristics of external environments, is the “biodiversity hypothesis”, which was triggered by studies revealing that children born and raised on farms have a low risk of allergy (7), mediated through environmental microorganisms (8). Haahtela et al. studied disease and exposure patterns in Finnish and Russian Karelia, a territory split between the two countries after World War II. The population is genetically similar and originally shared a natural environment, but was exposed to radically different societal structures and lifestyles. The allergy and asthma epidemic there became apparent in the mid-1960s. The Karelia Allergy Study 2002–2022 (9) showed that allergic conditions became far more common in Finland. The Russian population displayed richer gene–microbe networks with better-balanced immune regulatory circuits and lower allergy prevalence. In Finland, a biodiverse natural environment around the home was associated with lower allergy prevalence. Acting on the theory that lifestyle was causative, a nationwide Finnish Allergy Program was implemented from 2008 to 2018, endorsing immune tolerance, nature contact, and allergy health, with positive results (10). Subsequently, the Nature Step to Health 2022–2032 initiative was launched with the aim of preventing chronic diseases, nature loss, and climate change.

Barriers between the self and the environment, such as the skin, respiratory mucosa, and gastrointestinal mucosa, have evolved to protect health while allowing exchange of information and resources between organisms and their environment. Barrier function has developed in parallel with the immune system, under conditions that are very different from the rapidly changing ones induced by the industrial revolution over the last couple of centuries. The “epithelial barrier hypothesis” broadens the scope of substances that may influence immune balance beyond microbes alone. In this Research Topic, Sozener et al. argued that a myriad of potentially toxic substances are increasingly present in our world, compromising epithelial barriers. As a result, direct exposure of the immune system to exogenous allergens and microbes increases, leading to low-grade persistent inflammation (i.e., epitheliitis), production of alarmins, increased permeability, microbial translocation, dysbiosis, and consequent chronic type 2 inflammation.

Beyond these ‘wide’ hypotheses, additional perspectives included in this Research Topic illuminate aspects that cannot be neglected, along with their specific underlying mechanisms.

What effect might highly toxic exposures that occasionally occur on a large scale have? This is explored in the report by Shusterman and Simpson, who described the consequences of accidental exposure of bystander populations to highly toxic air pollutants. In two incidents—one in Bhopal, India, and the other in Dunsmuir, California, United States—separated by nearly a decade, exposure to cyanates led not only to acute and deadly toxic effects, but also to respiratory problems that remained chronic in many of those exposed. These events were linked by a common biological pathway involving the irritant ion-channel receptor TRPA1.

A less toxic, but much more frequent exposure was addressed by Smith and et al. Advanced Glycation End Products (AGEs) are compounds formed when sugars bind non-enzymatically to proteins, nucleic acids, or lipids. They can form *in vivo*, but they occur at much higher levels in the Western diet due to added sugars, protein dehydration, sterilization, and cooking methods. Dietary AGEs (dAGEs) can bind to the Receptor for Advanced Glycation End Products (RAGE), which is part of the endogenous threat-detection network. Epidemiological and biochemical data correlate increases in dAGEs with the rise in food allergies seen in several Western countries (11).

Shifting the focus from bacteria to arthropods, Retzinger and Retzinger put forward a hypothesis suggesting that allergies have increased due to the disruption of the ecological balance between primates and acarians (mites and ticks). The complete “Acari hypothesis” unfolded in six contributions, the last three of which are included in this collection. The hypothesis suggests that acarians potentiate, in the human host, the generation of IgE against elements of the acarian diet, which includes the majority of human allergens. Referring to the hygiene hypothesis, it argues that a major mechanism for deterring acarians—sweating—has been affected by modern hygienic practices. Furthermore, the hypothesis proposes that an acarian pattern-recognition receptor, possibly a fibrinogen-related protein, may elicit IgE responses following inoculation of the receptor in humans. The final installment introduces an additional player: the fungus *Malassezia*, whose feeding requirements are used to explain the increased predisposition of children to allergies. These are provocative ideas; however, further experimental and observational studies are needed to evaluate the relative contribution of this pathway to the allergy epidemic.

Two articles on “pre-asthma,” by Scadding et al., nicely demonstrated that our definitions and concepts of disease—and its boundaries—may fundamentally affect epidemiology, along with our potential for intervention. Prevention can be implemented at the population level, as in the Finnish asthma program, taking advantage of microbial interventions such as BCG or RSV vaccination (12, 13), early pathway blockade (e.g., through dupilumab) (14), or allergen-specific immunotherapy for allergic rhinitis (15). For secondary prevention, upper airway disease deserves better understanding and therapy.

Interestingly, a study by Lachover-Roth et al. investigated the acute epidemiological changes induced by the COVID pandemic. No differences in the development of atopic comorbidities were found between infants born before and during the pandemic. However, considerably higher airway hyperresponsiveness (AHR) was observed in infants born after restrictions were eased. It is possible that the increase in AHR resulted from the “bounce back” of many common cold viruses that had almost disappeared during lockdowns (16). Whether this will translate into longer-term epidemiological changes remains to be studied.

Considering the complete set of hypotheses included in this collection, one may observe that they can be complementary, and none excludes the others. Post–industrial revolution products—including hygiene-related ones—and environmental conditions may compromise the epithelial barrier, increasing pro-inflammatory signals. Diverse natural metagenomes may include important symbiotic microorganisms that are lost with decreasing biodiversity; both the composition and ecology of these communities are central drivers of human immunity. Mild respiratory infections associated with larger households may drive robust type 1 immunity, which is closely associated with eubiotic microbiomes (17). Higher-level organisms such as fungi and acarians are integral parts of the microbial environment and may contribute by driving immunity toward hyperreactive phenotypes. Disease classification and boundaries are also crucial for defining thresholds for intervention.

In the coming years, major efforts will be needed to translate the above concepts into improved protection of human health. Societies should be more aware of the exposome, including all the substances we are exposed to, the food we eat, and the air we breathe. Humanity would greatly gain from intensifying efforts to conserve—and bring closer to us—natural environments.
